# Maffucci syndrome complicated by giant chondrosarcoma in the left ankle with an *IDH1* R132C mutation: a case report

**DOI:** 10.1186/s12957-022-02686-z

**Published:** 2022-06-29

**Authors:** Haiyan Lv, Hantao Jiang, Minge Zhang, Huarong Luo, Zhenghua Hong, Hai Yang, Weiming Xu, Bo Shen, Wei Zhang, Hao Qiu, Rangteng Zhu

**Affiliations:** 1 Taizhou Hospital of Zhejiang Province affiliated to Wenzhou Medical University, 150 Ximen Road, Linhai, 317000 Zhejiang China; 2 DIAN Diagnostics, Hangzhou, 310058 Zhejiang China

**Keywords:** Maffucci syndrome, Chondrosarcoma, Isocitrate dehydrogenase

## Abstract

**Background:**

Maffucci syndrome (MS) is a rare, nonhereditary congenital mesodermal dysplasia characterized by multiple enchondromas and hemangiomas, associated with an increased risk of developing malignant tumors. Given their rarity, the pathogenesis of these tumors has not been clarified, and there is no standard treatment.

**Case presentation:**

We present a case of a 45-year-old man with MS to supplement the clinical manifestations and explore the molecular mechanism of MS. The patient underwent amputation surgery to inhibit tumor development and was diagnosed with MS with 1–2 grade giant chondrosarcoma in the left ankle. In addition, the whole exon analysis results revealed isocitrate dehydrogenase 1 (*IDH1*) R132C mutation in chondrosarcoma lesions but not in blood DNA.

**Conclusions:**

This case report showed MS complicated by giant chondrosarcoma in the left ankle with an *IDH1* R132C mutation, which is appropriate to monitor the development of MS pathology and other concomitant lesions.

**Supplementary Information:**

The online version contains supplementary material available at 10.1186/s12957-022-02686-z.

## Background

Maffucci syndrome (MS) is a rare cartilage dysplasia syndrome. In 1881, Angelo Maffucci first described a non-genetic disease characterized by multiple hemangiomas and endogenous chondroma during adolescence [1]. Clinically, MS is distinguished from Ollier disease (OD) by identifying soft-tissue vascular lesions accompanying MS but not OD. MS patients usually have asymmetric skeletal deformities and limb length differences in the first decade of life and may need surgery.

MS could occur in multiple races without gender differences or genetic predisposition. MS usually appears before the onset of puberty. However, 25% of the patients occurred at birth or within 1 year old, presenting with asymmetric leg contracture, swelling of the hands and feet, and occasionally fracture of the affected part. Endochondroma of MS grows slowly, with mild symptoms, local swelling, slight pain and tenderness, and pathological fractures. Often involving the iliac bone, metastasis is common in the lung [2].

Furthermore, additional tumors have been reported in MS patients with the disease progression, including lymphangiomas, glioma, acute myeloid leukemia, and ovarian fibrosarcoma [3, 4]. Recently, it has been revealed that individuals with MS and OD harbor somatic mosaicism of mutations in isocitrate dehydrogenase 1 (*IDH1*) or isocitrate dehydrogenase 2 (*IDH2*), as the crucial factors in the pathogenesis of these diseases [5]. At present, there are few studies on MS with chondrosarcoma and detailed gene analysis.

Therefore, this article reports a case of giant chondrosarcoma secondary to MS with a long medical history. Moreover, we conducted whole-exome sequencing of germline DNA and chondrosarcoma tissue to further explore the pathogenesis of MS.

## Case presentation

### History of present illness

A 45-year-old male patient was admitted to our hospital in October 2020 because of a lump in the left ankle for 20 years and enlarging over the last 2 years. From the appearance of the lump to the subsequent 18 years, there were no significant changes. However, the left ankle lump enlarged rapidly about 40 cm in diameter and then developed skin ulceration with apparent purulent discharge 2 years ago.

### History of past illness

At the age of 7, the patient accidentally found several soybean-sized soft lumps in two feet without pain and dysfunction, which was not paid attention to at that time. The patient developed multiple lumps in his limbs in the next few years. When the patient was 19 years old, he was finally diagnosed with MS for the extremities with multiple angiomatosis and enchondromas and underwent resection surgery in our hospital. However, the symptoms reoccurred at the original resection site only 2 years later and resection surgery again. Confusingly, reoccurrence happened soon after the second resection surgery.

### Physical and imaging examination

**Fig. 1:**
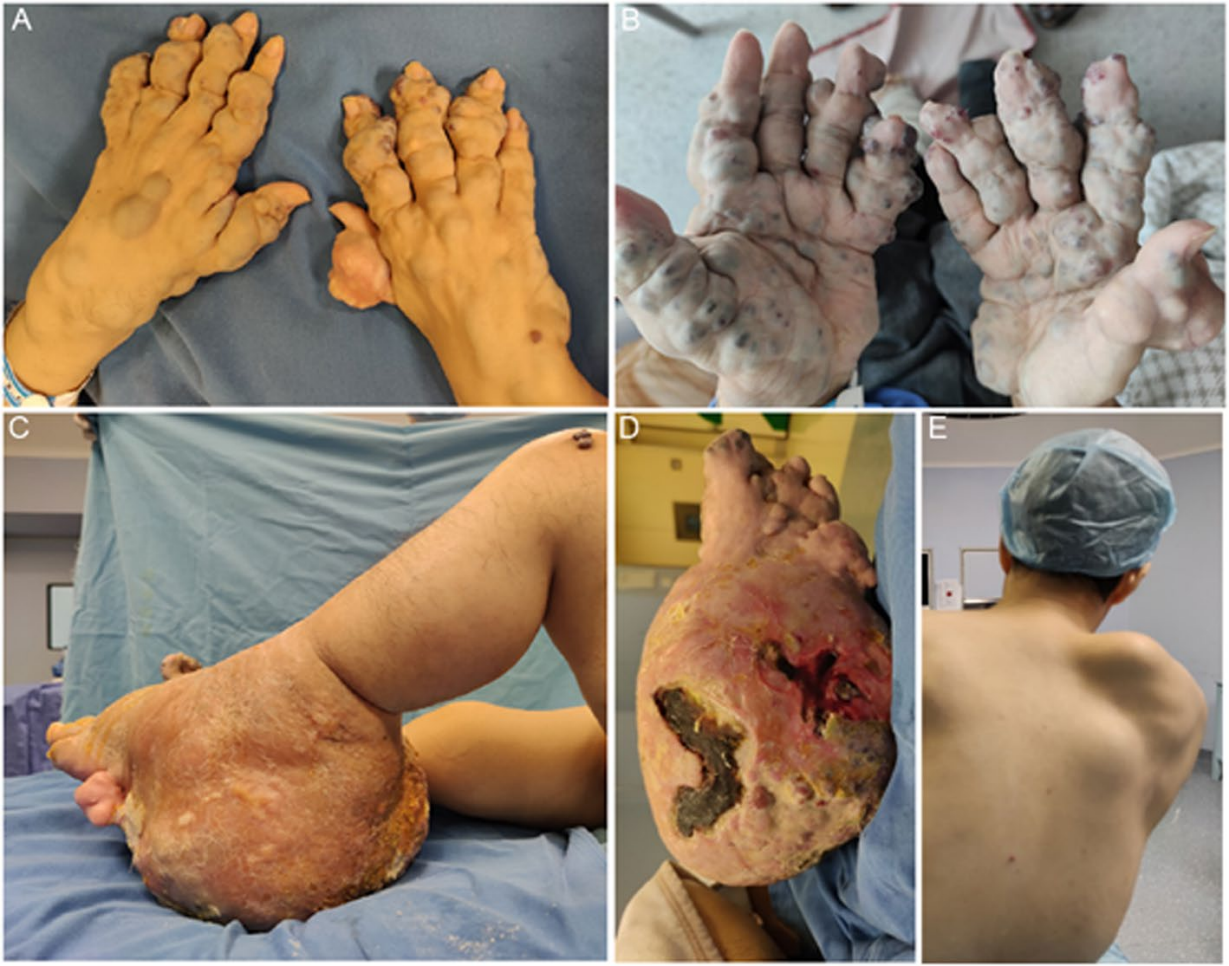
Physical examination. **A**, **B** Multiple angiomatoses and enchondromas in the extremities. **C**, **D** A giant lump in the left ankle. **E** A giant lump on the right scapula

After the patient was admitted in 2020, the physical examination showed multiple angiomatoses and enchondromas in the extremities (Fig. 1A, B), and a giant lump of about 35 cm × 30 cm × 30 cm in the left ankle, with local skin ulceration and evident purulent secretion discharge (Fig. 1C, D). There was a big lump on the right scapula without tenderness or percussion pain (Fig. 1E).

**Fig. 2:**
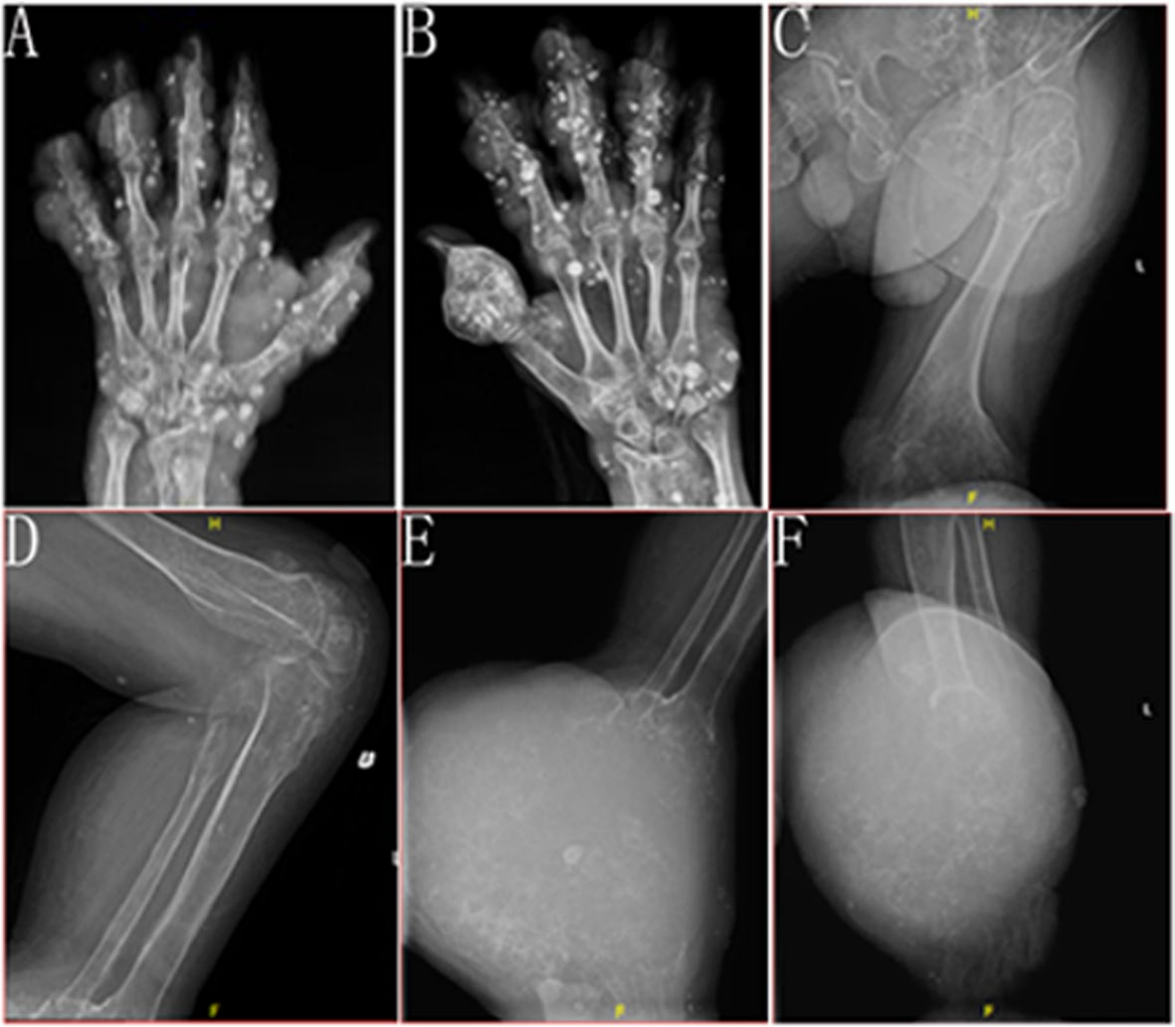
CT examination. **A**, **B** Spherical expansive bone destruction in the right thumb and multiple phleboliths in the hands and wrists. **C** Bone density and morphological changes of the left ilium and left femur. **D** Multiple venous stones around the left knee joint. **E**, **F** Spherical expansive bone destruction with multiple calcifications at the left tarsal

**Fig. 3:**
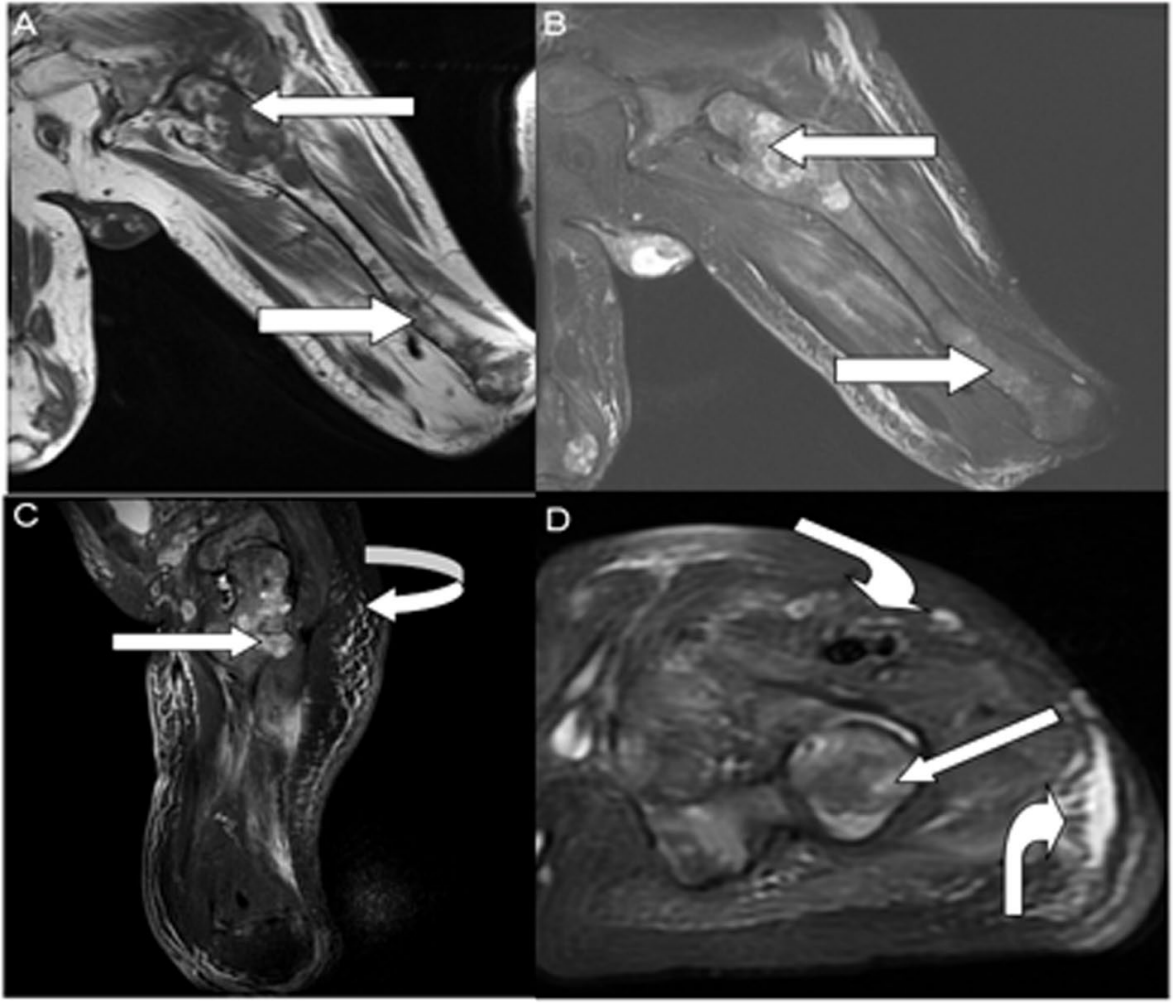
Preoperative imaging studies. **A** T1WI, the bone marrow cavity of the left femoris was dilated and irregular in shape, with multiple irregular patchy and nodular hyposignal foci (straight arrow). **B**, **D** The coronal, sagittal, and cross-sectional views of the STIR show the appearance of the lesion. T1WI shows the lesion with low-signal intensity. Inhomogeneous high signal, unclear boundary and adjacent cortical destruction in STIR ( straight arrow), surrounding soft tissue swelling, and exudation (curved arrow)

X-ray examination of both hands revealed changes in bone morphology and density of both hands, including spherical expansive bone destruction in the right thumb and multiple phleboliths in the hands and wrists (Fig. 2A, B). In addition, we found bone density and morphological changes in the left ilium and left femur (Fig. 2C), the flexion deformity of the left knee joint, and multiple venous stones around the left knee joint (Fig. 2D). Moreover, X-ray examination showed local spherical expansive bone destruction with multiple calcifications at the left tarsal, multiple chondromatosis with bone deformity, and multiple hemangiomas (Fig. 2E, F). In addition, magnetic resonance imaging (MRI) was performed on the patient’s left femur to determine the amputation plane (Fig. 3). We found that the endogenous chondroma has involved the whole length of the left femur without chondrosarcoma; multiple patchy and nodular abnormal signals were observed in the left femur bone marrow cavity with low T1WI and high STIR signals. MRI examination also showed the swelling and exudation of the soft tissue around the femur surrounding soft tissue.

### Oncological management

The patient was confirmed as having chondrosarcoma by biopsy prior to surgery. Although there were no typical appearances of MRI that demonstrated sarcoma metastasis, the patient’s knee has become stiff and nonfunctional. Therefore, the patient eventually underwent above-the-knee amputation. The tumor edge was saved 23 cm from the tibial tubercle. Therefore, surgical margins greater than 23 cm from the tumor meet the treatment criteria for wide excision. During the operation, clear fluid flowed out of the marrow cavity of the femur, and muscle edema was observed. Regrettably, the aspirator accidentally removed the liquid, leaving no specimen left. The surgical incision was sutured after intraoperative pathology confirmed the negative surgical margins. Most conventional chondrosarcomas have low metastatic potential and are both radiation and chemotherapy resistant, so the patient did not receive radiotherapy and chemotherapy after surgery.

### Pathological examination

**Fig. 4:**
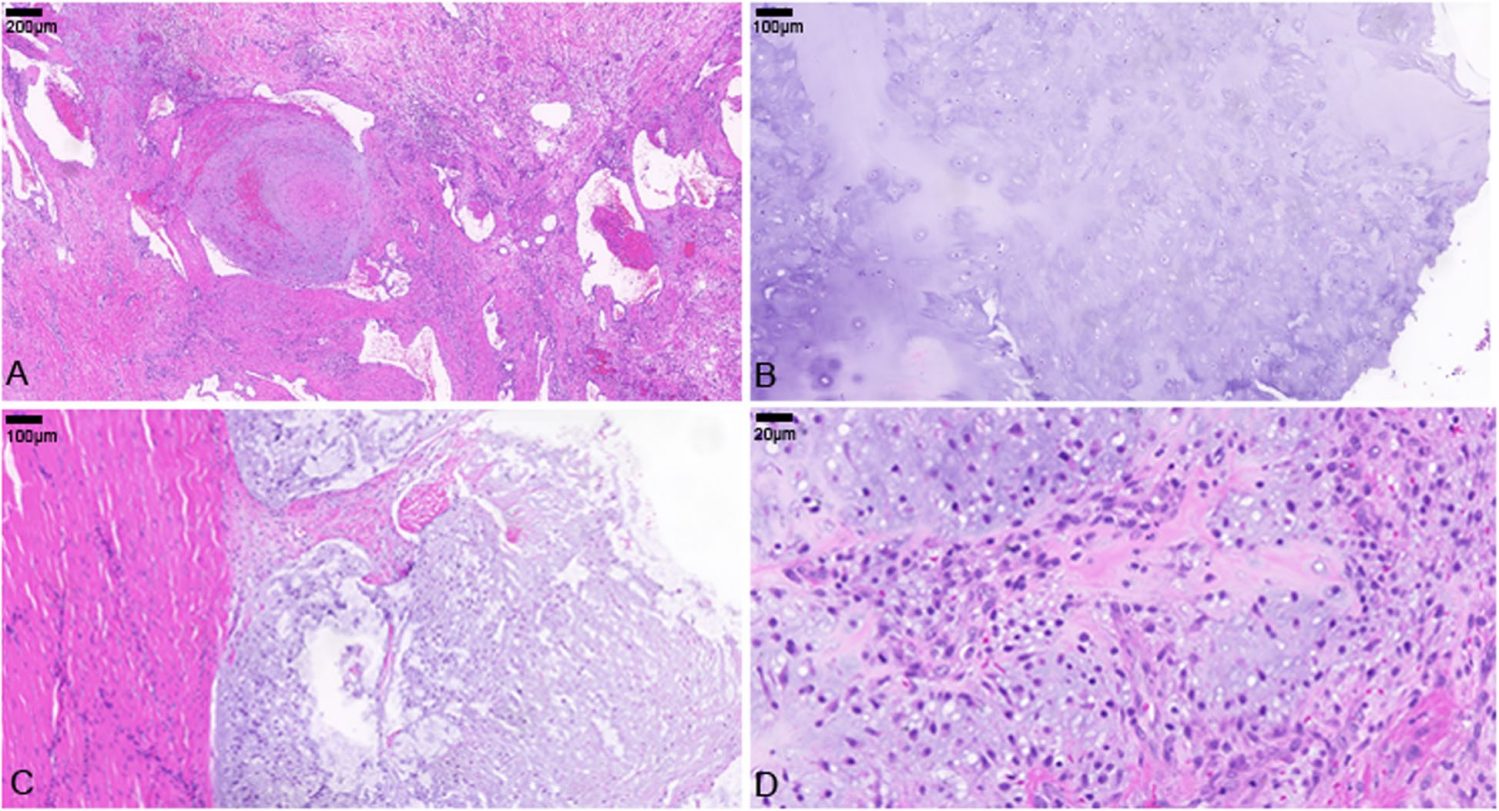
Microscopical features of the pathological sections with hematoxylin–eosin staining. **A** Hemangioma, × 5. **B** Endochondroma, × 10. **C**, **D** Chondrosarcoma, × 10 and × 40, respectively

The resected specimen is shown in the supplementary material (Fig. S1A), and the tumors were apparent in Fig. S1B. The chondrosarcoma of the left ankle was assessed as grades 1–2 by pathological diagnosis. Multiple cavernous hemangiomas with thrombosis were in the left lower limb (Fig. 4A). Enchondroma was observed in the tibia. The cells were loose, with less atypia (Fig. 4B). Besides, chondrosarcoma invades surrounding soft tissues, and necrosis was seen in some areas (Fig. 4C). Chondrosarcoma cells with prominent atypia and visible nuclear fission (Fig. 4D).

### Genetic testing

DNA was extracted from the peripheral blood and chondrosarcoma tissue and performed exome sequencing. Genomic DNA from FFPE tumor samples and paired blood samples were extracted by QIAamp DNA FFPE Tissue KIT and QIAamp DNA Blood Midi KIT (Qiagen, Dusseldorf, Germany) following the manufacturer’s instructions, respectively. The QIAseq FX DNA Library UDI Kit (Qiagen, Dusseldorf, Germany) was used to generate indexed whole genome libraries from 50-ng genomic DNA according to handbook protocols. Hybridization capture was performed using the IDT xGen Exome Research Panel v1.0 (Integrated DNA Technologies, Coralville, IA, USA) according to the manufacturer’s protocol. Finally, whole exome libraries were quantified by qPCR using the QIAseq Library Quant Assay Kit on a QIAquant 384 instrument (Qiagen, Dusseldorf, Germany). Paired-end sequencing (2 × 150 bp) was performed on an Illumina NovaSeq 6000 sequencing instrument (Illumina, San Diego, CA, USA). Then, the data were mapped to the human reference genome (GRCh37/hg19) using the Burrows-Wheeler Aligner (BWA) [6]. Picard was used to removing duplicated sequence reads. The Genome Analysis Toolkit (GATK) [7] HaplotypeCaller and MuTect [8] were applied to identify candidate germline and somatic single-nucleotide variants (SNVs), respectively. SNV annotation was performed using ANNOVAR [9], and somatic copy number alterations were identified using CNVkit [10]. Somatic structural variations were detected using DELLY [11].

Finally, germline mutations of the tumor susceptibility gene were not discovered. However, a somatic missense mutation was identified in *IDH1* gene compared with germline DNA variants (nucleotide variant: NM_005896.4: c.394 C > T, amino acid variant: NP_005887.2: p.Arg132Cys, rsID:rs121913499), which the mutation ratio was 14.71%. It has been recorded as pathogenic and likely pathogenic in ClinVar (Variation ID: 375,891). The frequency of this locus in normal East Asian populations has not been reported. Thus, *IDH1* R132C, in our case classified as pathogenic.

### Follow-up

We have followed up with the patient for 15 months, the wound is healing as well, and no obvious recurrence was observed at present (Fig. S1C). Hemangiomas and chondroma in other sites had no progress. In addition, the patient has been fitted with a prosthesis and can walk on the ground.

## Discussion

This article reports a case of giant chondrosarcoma secondary to MS. The patient had a 39-year history of MS and a 2-year history of chondrosarcoma in the left ankle. Due to the large size (40 cm in diameter) and the malignant of the left ankle chondrosarcoma, we performed an amputation surgery to inhibit the tumor development. For the first time, we conducted whole-exome sequencing of germline DNA and chondrosarcoma tissue in MS patient, revealing *IDH1* R132C somatic mutation in chondrosarcoma lesions but not in blood DNA.

Enchondroma is a benign intramedullary chondroma that could occur at any position in MS patients. In our study, it develops on the bilateral scapula, the right fifth rib, both ilia, the left pubic branch, the left femur, the upper and lower ends of the tibia and fibula, and the metacarpal and phalanx. The risk of enchondroma developing into chondrosarcoma is approximately 25 to 30% in patients with MS, usually younger than primary chondrosarcoma patients [12]. Malignant changes frequently occur after 40 years old and could occur in any part of the affected bone. The pelvic and upper limb bones are more prone to malignant changes. Skull base chondrosarcoma accounted for 5 to 10% of all MS reports [13]. To the best of our knowledge, the female patient with MS developed chondrosarcoma of the femur at the age of 32 and a biliary adenocarcinoma at the age of 44 was described as early as 1987 [14]. Rarely it has been reported a 34-year-old man with tracheal chondrosarcoma and a 39-year-old woman with nasal cavity chondrosarcoma in MS patients [15, 16]. The patient in our study deteriorated to chondrosarcoma in the left ankle around 43 years old. It is remarkable that our MS patient complicated with chondrosarcoma in the ankle is very large and fast-growing, which is extremely rare among the MS reports.

A hemangioma can be seen in many parts, and 8.5% of individuals with MS will develop a vascular malignancy [17]. According to the National Organization for Rare Diseases, vascular abnormalities in MS patients generally occur in children ages 4–5 years, most commonly in the hands. Among individuals with MS, about half had unilateral vascular anomalies (47.2%), and 63% (63.8%) had vascular anomalies in the upper extremity, while 52.7% had them in the lower extremity, and only two individuals (5.5%) had visceral involvement [17]. In this case, multiple hemangiomas in both hands were observed.

We found *IDH1* R132C somatic mutation in chondrosarcoma lesions in the present case. The *IDH1* R132C has been detected previously in multiple tumor tissues. The most common missense mutation for the *IDH1* Arg132 codon is histidine (*IDH1* R132H). Others are also reported, such as cysteine, serine, glycine, leucine, or isoleucine [18, 19]. All these variants (*IDH1*(NM_005896.4)_ex4 c.395G > C (p.Arg132Pro), c.395G > T (p.Arg132Leu), c.395G > A (p.Arg132His), c.394C > A (p.Arg132Ser), and c.394C > G (p.Arg132Gly)) were all annotated as pathogenic or likely pathogenic in the ClinVar database (Variation ID: 375,890, 375,889, 156,444, 375,893, 375,892). The *IDH1* R132C variant has been recorded in a COSMIC database for the blood and lymphatic system tumors, bone tumors, central nervous system tumors, biliary tumors, and other tumors (Genomic Mutation ID: COSV61615256). Variant R132C is also recorded in the CIViC database (Allele Registry ID: CA16602374).

*IDH1* Arg132 residue locates at the enzyme affinity site for the substrate, which can catalyze isocitrate to produce α-ketoglutarate (α-KG). It is highly conserved during evolution and functionally important. Variant R132C is a gain-of-function mutation, changing the catalytic activity of the enzyme and resulting in the conversion of α-ketoglutarate (α-KG) to 2-hydroxyglutarate (2-HG) [20, 21]. In normal cells, 2-HG is usually present at a very low level. However, excessive 2-HG may promote cell transformation by changing the redox state of cells or leading to metabolic and epigenetic changes [21, 22].

Literature showed that 77% of MS patients carry *IDH1* or *IDH2* mutations, which are often present in malignant tissues [23]. Recently, somatic mosaic *IDH1* R132C variant in DNA derived from hemangioma tissue but absent in DNA derived from the blood was identified in an adult male with MS [24]. A 39-year-old MS woman together with intrahepatic cholangiocarcinoma (IHCC) showed *IDH1* R132C mutation in the tumor tissue but not in the normal liver tissue and peripheral blood [4]. Furthermore, a MS patient with jugular foramen chondrosarcoma and pituitary adenoma revealed the same *IDH1* R132C mutation in both tumors by Sanger sequencing [25]. Similarly, another report had shown common *IDH1* R132C mutations in sellar, brainstem, and skull base tumors in MS patients; no *IDH2* mutations were detected; and both *IDH1* and *IDH2* were wild types in blood DNA by Sanger sequencing [26]. However, no studies have shown that the same mutations are present in non-lesioned tissue or blood system. Our case revealed that the *IDH1* R132C mutation was only present in the ankle chondrosarcoma tissue but not in the blood of this patient, and the presence of the mutation in the enchondroma/hemangioma tissue of the patient remains to be confirmed.

Consistently, the multiple enchondromas in OD and MS are the consequence of a post-zygotic germline mutation resulting in a mosaic distribution of mutant cells [27]. However, the culprit gene (S) is still elusive. Since *IDH1/IDH2* mutations were detected in both isolated chondromas and tumors removed from patients with multiple lesions (OD/MS). Interestingly, some speculate that these mutations represent the early most synonymous genetic events and explain the onset of the disease. The detection of the same *IDH1/IDH2* mutation in somatic tissues of the same patient but with low frequency will provide clear evidence for this. However, we demonstrated no mutation in the blood and could not prove mutations in non-invasive tissues in our case, which is difficult for patients with the mosaic disease.

Additionally, the same spectrum of mutations in *IDH1* and *IDH2* also leads to other malignancies. *IDH1* and *IDH2* mutations were first found in adult glioblastoma multiforme (GBM) patients [28]. Recurrent somatic mutations in *IDH1* and *IDH2* occurred in about 80% of patients with grade II–III GBM and secondary GBM, and it is an early event of GBM progression [29, 30]. *IDH* mutations were also found in 10–20% of patients with acute myeloid leukemia (AML), with a low incidence in other cancers; the majority of these lesions involve arginine (R) residue mutations in *IDH1* codon 132 (*IDH1* R132) and residues 140 and 172 of *IDH2* (*IDH2* R140 and *IDH2* R172) [31, 32]. These findings suggested that somatic heterozygous mutations in *IDH1* or *IDH2* are also crucial in developing some malignant tumors. However, exactly how these variants contribute to this broad spectrum of diseases remains unclear.

The treatment of MS aims to alleviate the clinical symptoms of patients and detect malignant lesions early. Surgery is the primary treatment for bone and vascular diseases, and amputation should be considered for patients with severely affected functions or malignant changes. The essential biochemical indicator of *IDH1* and *IDH2* mutations in the peripheral blood is the abnormal increase of the 2-HG level [33], which may be a sensitive and specific predictor. The first oral *IDH1* inhibitor Ivosidenib (AG-120) has been shown to reduce 2-HG levels and induce cell differentiation in vitro and in vivo [34]. More recently, sirolimus has been reported in two studies on the treatment of vascular lesions–one was unsuccessful; however, the other was successful when combined with surgical treatment [35, 36]. Nevertheless, no studies have shown that molecular therapy has successfully applied to MS patients, and we did not attempt adjuvant therapy.

The prognostic challenge of MS not only comes from the skeletal deformities and secondary limb length differences caused by itself but as well as the potential risk of malignant transformation into chondrosarcoma. Researchers conducted on 7 MS patients and showed that no patient died of skeletal sarcoma, but with non-skeletal malignancies in the study [14]. Therefore, in addition to routine clinical care for MS patients, clinicians should actively detect the development of endogenous chondromas in various parts and do well in tumor detection outside the bone tissue, such as the brain and abdomen.

## Conclusion

This is a case report of MS complicated by giant chondrosarcoma in the left ankle with an *IDH1* R132C mutation. To reduce missed diagnosis or early detection of concomitant lesions, imaging examination and follow-up of other organs are essential. Considering that no subsequent shared genetic events were identified in the chondrosarcoma from our limited analyses, it is critical to conduct a further comprehensive investigation to discover *IDH1*-associated tumorigenesis.

## Supplementary Information

**Additional file 1:** **Fig. S1.** The resected specimen and surgical site of the patient. (A) The resected specimen. (B) Apparent tumors in the resected specimen. (C) The surgical site of the patient.

## Data Availability

The datasets used and/or analyzed during the current study are available from the corresponding author on reasonable request.

## Abbreviations

MS: Maffucci syndrome
OD: Ollier disease
*IDH*: Isocitrate dehydrogenase
MRI: Magnetic resonance imaging
α-KG: α-Ketoglutarate
2-HG: 2-Hydroxyglutaric acid
NADPH: Nicotinamide adenine dinucleotide phosphate
GSH: Glutathione
GBM: Secondary glioblastoma
AML: Acute myeloid leukemia
R: Arginine
