# Supplementary figures and images for: Maffucci syndrome complicated by giant chondrosarcoma in the left ankle with an IDH1 R132C mutation: a case report
Source: World J Surg Oncol. 2022 Jun 29;20:218. doi: 10.1186/s12957-022-02686-z (PMC9241289; doi:10.1186/s12957-022-02686-z)

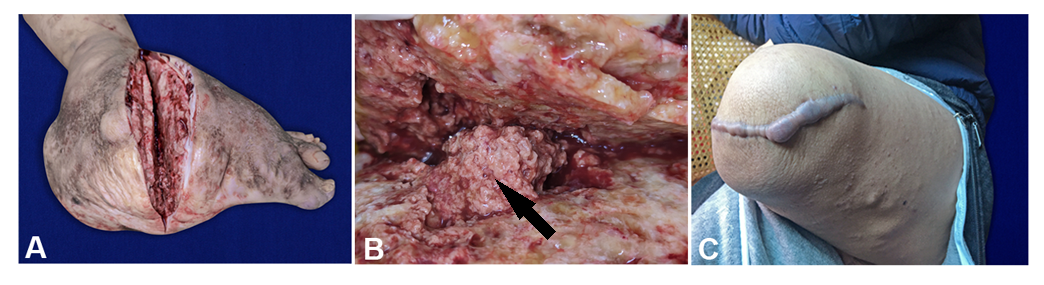

Supplement: Supplementary file 1 — Additional file 1: Fig. S1. The resected specimen and surgical site of the patient. (A) The resected specimen. (B) Apparent tumors in the resected specimen. (C) The surgical site of the patient. [file 12957_2022_2686_MOESM1_ESM.tif]
